# Underwater hyperspectral imaging as an *in situ* taxonomic tool for deep-sea megafauna

**DOI:** 10.1038/s41598-018-31261-4

**Published:** 2018-08-27

**Authors:** Ines Dumke, Autun Purser, Yann Marcon, Stein M. Nornes, Geir Johnsen, Martin Ludvigsen, Fredrik Søreide

**Affiliations:** 1 Department of Marine Technology, Norwegian University of Science and Technology (NTNU), Trondheim, Norway; 2 Alfred Wegener Institute, Helmholtz Centre for Polar and Marine Research, Bremerhaven, Germany; 3 MARUM, Center for Marine Environmental Sciences, Bremen, Germany; 4 Centre for Autonomous Marine Operations and Systems, Department of Biology, Norwegian University of Science and Technology (NTNU), Trondheim, Norway; 5 University Centre in Svalbard (UNIS), Longyearbyen, Svalbard Norway; 6 Present Address: GEOMAR Helmholtz Centre for Ocean Research Kiel, Kiel, Germany

## Abstract

Identification of benthic megafauna is commonly based on analysis of physical samples or imagery acquired by cameras mounted on underwater platforms. Physical collection of samples is difficult, particularly from the deep sea, and identification of taxonomic morphotypes from imagery depends on resolution and investigator experience. Here, we show how an Underwater Hyperspectral Imager (UHI) can be used as an alternative *in situ* taxonomic tool for benthic megafauna. A UHI provides a much higher spectral resolution than standard RGB imagery, allowing marine organisms to be identified based on specific optical fingerprints. A set of reference spectra from identified organisms is established and supervised classification performed to identify benthic megafauna semi-autonomously. The UHI data provide an increased detection rate for small megafauna difficult to resolve in standard RGB imagery. In addition, seafloor anomalies with distinct spectral signatures are also detectable. In the region investigated, sediment anomalies (spectral reflectance minimum at ~675 nm) unclear in RGB imagery were indicative of chlorophyll *a* on the seafloor. Underwater hyperspectral imaging therefore has a great potential in seafloor habitat mapping and monitoring, with areas of application ranging from shallow coastal areas to the deep sea.

## Introduction

Traditionally, assessment of the megafauna inhabiting a region of deep-sea seafloor has been determined either from direct, invasive physical sampling using box corer, trawl^1,2^ or Remotely Operated Vehicles (ROVs)^3–5^, or by remotely imaging the seafloor with video or still camera systems mounted on towed platforms^6–8^, ROVs^3,9–11^ or Autonomous Underwater Vehicles (AUVs)^12^. Over the last decade, alternative optical methods have been developed for identification and characterization of organisms. These methods are based on hyperspectral imaging, i.e., the acquisition of images integrating hundreds of contiguous colour bands (wavelengths), such that each image pixel contains a full spectrum, typically with 1 nm spectral resolution^13^. Each image pixel spectrum describes the percentage of light reflected by the material of that image pixel for each wavelength. The reflectance spectra represent so-called optical fingerprints that are specific to the object of interest (OOI).

To study biological organisms underwater, the visible range of the electromagnetic solar spectrum (400–700 nm) is typically used, due to the strong attenuation of light in the ultraviolet (<400 nm) and infrared (>700 nm) regions of the electromagnetic spectrum^14–17^. However, some studies also extended reflectance measurements to the near-infrared region (up to 900 nm)^18–21^. The high spectral resolution current hyperspectral imaging systems can achieve (up to 1 nm) and extended spectral range therefore provide a greater volume of colour information than conventional RGB imagery, facilitating the detection, and potentially aiding in the identification of organisms in seafloor images.

Previous studies have focused on the detection of pigmented organisms based on pigment-specific spectral characteristics, using a combination of pigment extraction from plants, algae, and fauna, and subsequent analysis by high performance liquid chromatography, followed by measurements of absorption and reflectance spectra^16,22–24^. As reflectance and absorption spectra are inversely related to each other, absorption spectra can be used to verify reflectance signatures from organisms^16^. Applications have included *in vitro* studies of biofilms^18^ and *in vivo* analysis of sponges^16^, cold-water corals^21,23^, micro- and macroalgae^14^, and coralline algae^24^. These results have shown that hyperspectral imaging represents a useful bio-optical taxonomic tool for *in vivo* identification of biological organisms.

During the last eight years, hyperspectral studies of marine organisms and other seafloor OOIs have been conducted *in situ*, using diver-operated Underwater Hyperspectral Imagers (UHIs) in shallow waters^17,20^, as well as by mounting UHIs on mechanical sledges^15,16,19^, on ROVs^17,24–26^, and on AUVs^27^. These studies indicate the promising potential for underwater hyperspectral imaging to greatly aid in the mapping and characterization of different seafloor OOIs *in situ*, including microphytobenthos, kelp forests, coralline red algae, and coral reefs^17,19,20,24^, as well as identifying and characterizing seafloor mineral deposits and geological structures^26,27^.

By using UHIs on ROVs instead of diver-operated or sledge-based UHIs, seafloor areas of up to 1000 m^2^ may be surveyed in water depths of up to 6000 m. At such depths, sampling of animals for accurate taxonomic identification is generally difficult and time-consuming. Identification of fauna from these remote regions can therefore be very problematic, given the few available physical samples. Whilst standard RGB-based optical imaging methods may collect large datasets, interpretation of these data is not straightforward, with groups of experts potentially interpreting abundances in images differently^7^. At present, there is a tendency to differentiate fauna to a fairly broad taxonomic ‘morphotype’, given the great disparity that can result from even multiple experts investigating a deep-sea fauna image dataset^7,11^. Attempts at using machine learning algorithms to identify fauna vary in success with ecosystem and fauna^28^.

By identifying fauna by their specific spectral responses, underwater hyperspectral imaging may provide *in situ* taxonomic identifications of benthic communities in a more standardized fashion than is currently achievable by human researchers or algorithms applied to human-identified training sets^7^. This is particularly useful in areas where long-term monitoring of seafloor community structure may have important management applications, such as in the vicinity of oil and gas drilling^10^, deep sea fishing^29^, or deep sea mineral extraction sites^11,30^.

In this study, we show that underwater hyperspectral imaging can be used as a non-invasive, *in situ* taxonomic tool for benthic megafauna in deep-water habitats. Megafauna may be identified based on their spectral characteristics in UHI image data without the need for physical sampling of animals. In addition, we show that the approach is also useful for detection and mapping of sediment anomalies, including potential seafloor biomass accumulations of phytodetritus, as indicated by elevated chlorophyll *a*-like signatures.

## Results

**Figure 1:**
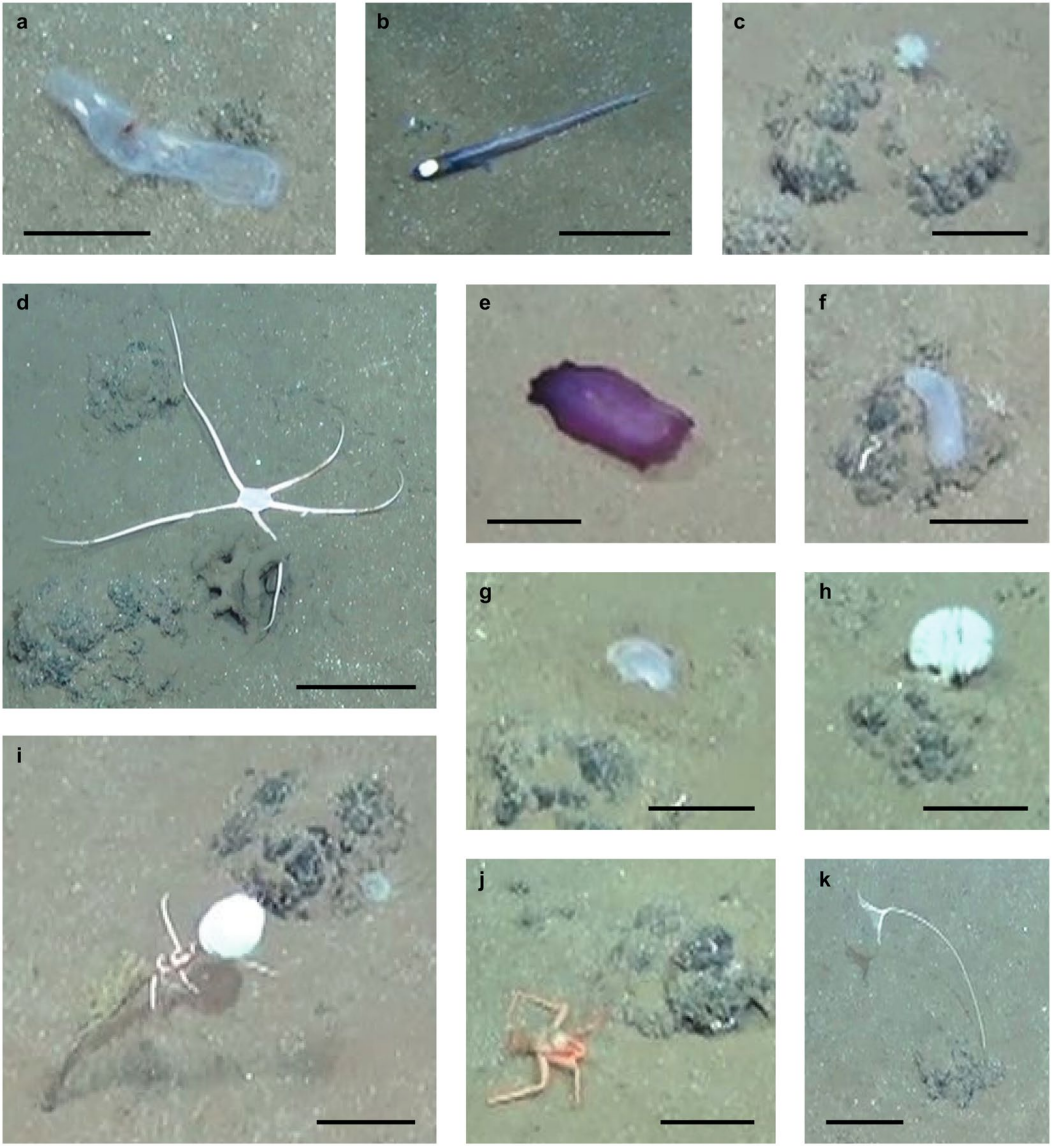
Images from HD video frames for different mobile and sessile megafauna observed in the study area. All scale bars measure 6.5 cm. (**a**) dead salp, (**b**) bony fish (*Ipnops* sp.), (**c**) Demosponge (round) on manganese nodule, (**d**) ophiuroid, (**e**) holothurian (*Paelopatides* sp.), (**f**) holothurian (Synallactidae) with a sessile polychaete on the left side and a small coral on the right side, (**g**) Demosponge (disc), (**h**) Hexactinellid, (**i**) stalked sponge with a green coral attached to the stalk and an ophiuroid wrapped around it, (**j**) crustacean (*Probeebei mirabilis*), (**k**) stalked crinoid. Images: ROV KIEL 6000, ROV Team GEOMAR.

Seabed OOIs, i.e., megafauna individuals and sediment anomalies, were detected across all 11 ROV transects analysed, with all transects containing both megafauna and sediment anomalies. A total of 30 OOIs were identified based on the video data and previous sampling, and included 28 mobile and sessile megafauna across nine of the 11 transects. Up to four fauna categories with 1–2 individuals per category were observed on each of these transects, including sponges (Demospongiae and Hexactinellida; Fig. 1c,g,h), ophiuroids (Ophiuroidea; Fig. 1d), holothurians (*Paelopatides* sp. and Synallactidae; Fig. 1e,f), corals (Fig. 1f,i), polychaetes (Fig. 1f), crustaceans (*Probeebei mirabilis*; Fig. 1j), dead salps (Fig. 1a), as well as a crinoid (Crinoidea; Fig. 1k) and an isopod (Munnopsidae). One bony fish (*Ipnops* sp.; Fig. 1b) was also observed. Megafauna individuals detected in the video data measured about 2–15 cm in length (average 6 cm). Although more animals were visible in the video data, they were not clearly resolved due to their small sizes (<2 cm), and hence identification was not possible. In addition, two anomalous sediment patches of a lighter colouration than the surrounding seafloor, likely representing recently exposed sediments from below the active surface sediment layer, were observed.

**Figure 2:**
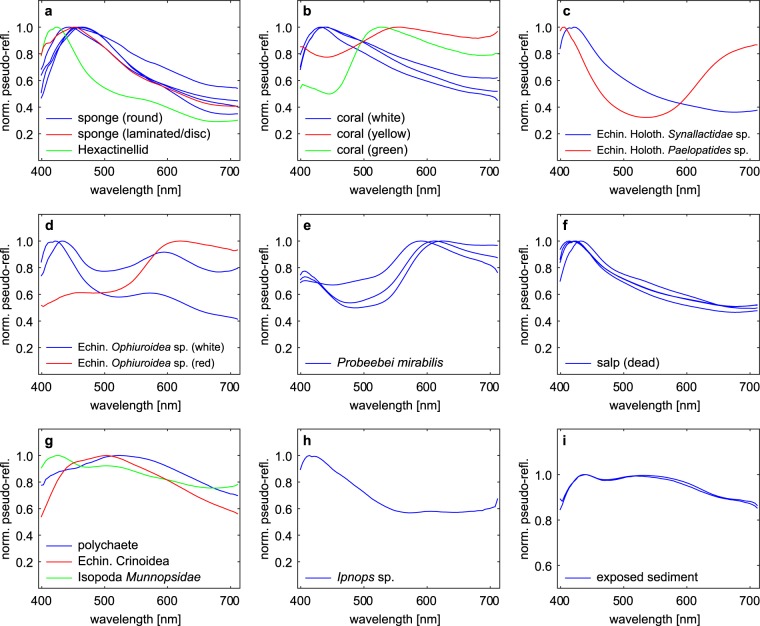
Average pseudo-reflectance spectra (normalized to their maximum value) of the 30 OOIs identified from the video data, including different mobile and sessile megafauna (**a**–**h**), and sediment exposed from below the surface layer (**i**). These spectra were used as reference for identification of additional OOIs. The smooth appearance of the spectra is due to smoothing by a moving average filter (11 band window) during processing, and averaging over OOI surface areas of 20 to 600 pixels.

The 30 OOIs identified from the video data were clearly visible in the UHI images and exhibited spectral signatures that were distinct from the background spectra of sediment and manganese nodules, which were relatively stable throughout the transects. The OOI spectra also differed between the imaged fauna morphotypes, whilst the spectral signatures of different individuals of the same morphotypes were similar (Fig. 2). For example, the three crustaceans (*Probeebei mirabilis*; Fig. 2e) were characterized by very similar spectral responses. Five dead salps, common seafloor ecosystem components during the research cruise^31^, also showed similar spectral responses (Fig. 2f). In the case of corals, spectral signatures of surveyed green and yellow corals did not match those of three surveyed white corals (Fig. 2b). Similarly, two white ophiuroids exhibited a different spectral response than a red ophiuroid (Fig. 2d). Two observed holothurians, which were of different species (Synallactidae and *Paelopatides* sp.), also had different spectral signatures, differing most markedly in the red part of the spectrum (600–710 nm; Fig. 2c).

**Figure 3:**
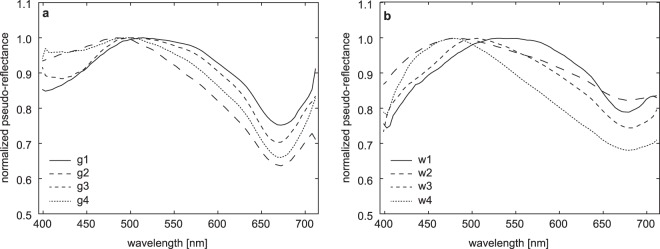
Examples of normalized pseudo-reflectance spectra of the anomalous green spots (**a**) and white spots (**b**). Green spots g1-g4 are shown in Fig. 4, white spots w1-w4 are marked in Fig. 5. Both green spots and white spots are characterized by a prominent intensity minimum around 675 nm, resembling reflectance dips of chlorophyll *a* or its degraded form, phaeophorbide *a* and/or phaeophytin *a*.

**Figure 4:**
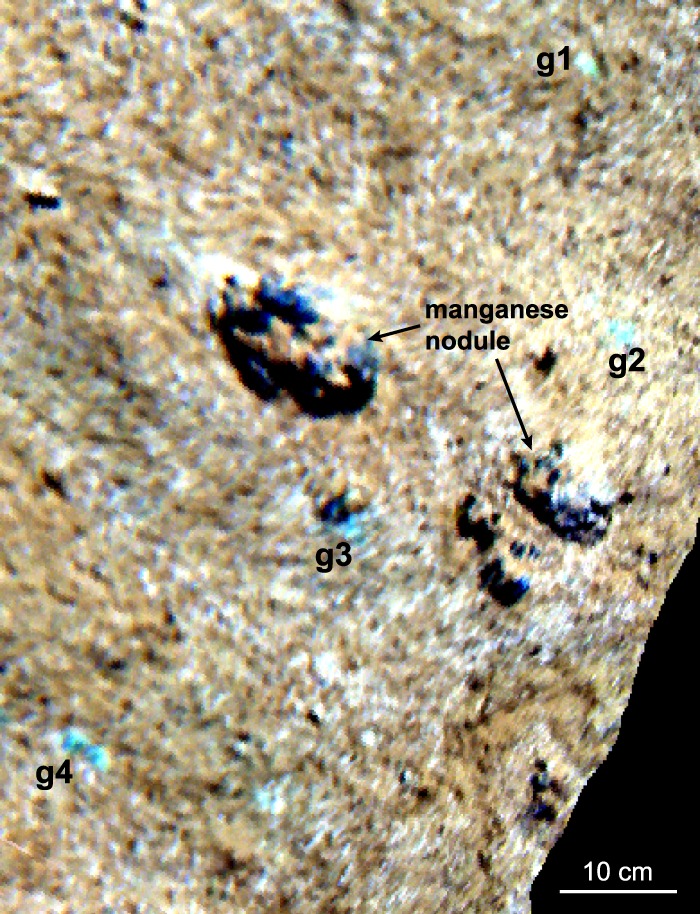
Processed UHI data (pseudo-reflectance) in pseudo-RGB colours (R: 645 nm, G: 571 nm, B: 473 nm), showing the spectral anomalies termed “green spots”. Spectra of the green spots marked g1-g4 are shown in Fig. 3a.

**Figure 5:**
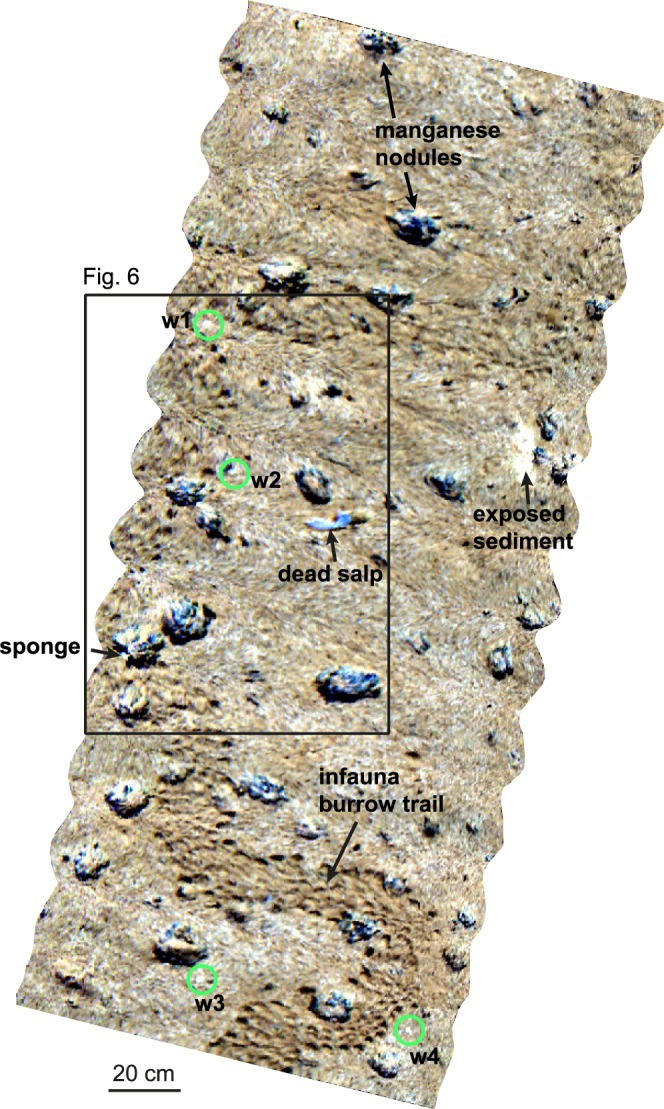
A 2.9 m long UHI transect (in pseudo-RGB with R: 645 nm, G: 571 nm, B: 473 nm) across manganese nodules, a dead salp, a sponge and an infauna burrow trail. Annotations w1-w4 mark the white spots belonging to the spectral responses shown in Fig. 3b.

In addition to the 30 OOIs identified from the video data, 48 further OOIs were detected in the UHI images of each transect, with numbers ranging between two and six additional OOIs per transect. The calculated spectral contrast angle, which was used to compare the spectra of these 48 OOIs to the 30 reference spectra, varied between 0.63° and 30° in general and between 0.63° and 3.03° for the smallest angle (1.75° on average). Based on the smallest spectral contrast angle, 13 OOIs were associated with megafauna, in particular white corals and polychaetes (Table 1). The remaining 35 OOIs represented sediment anomalies. Six of these were reminiscent of the exposed deeper sediment patches, while the other 29 OOIs had spectral signatures characterized by a distinct intensity minimum around 668–680 nm (Fig. 3) that was not observed in the spectra belonging to the exposed deeper sediment patches. These OOIs were roughly circular spots with maximum diameters of 2 cm that appeared either green or white in the UHI images displayed in pseudo-RGB (R: 645 nm, G: 571 nm, B: 473 nm; Figs 4 and 5). Along each transect, at least one of these anomalies was observed in the UHI images, while they were barely or not visible in the video data. Due to the characteristic intensity minimum, these OOIs were assigned to two new spectral categories termed “green spot” and “white spot” (Table 1). Spectral signatures were relatively similar for both the green and the white spots, although pseudo-reflectance intensities were slightly higher for the white spots, especially for wavelengths >650 nm (Fig. 3).

**Table 1:** Overview of OOI categories and classification results.

Category	Taxonomy	Training spectra			SVM classification results
		from video/samples	from spectral anomalies	total training spectra	
**Megafauna**					
Sponge (round)	Porifera, Demospongiae sp1	4		4	8
Sponge (disc)	Porifera, Demospongiae sp2	1		1	1
Glass sponge	Porifera, Hexactinellida	1		1	1
Coral (white)	?	3	8	11	39
Coral (yellow)	?	1		1	1
Coral (green)	?	1		1	1
Holothurian Synallactidae	Echinodermata, Holothuroidea, Synallactidae	1		1	1
Holothurian *Paelopatides*	Echinodermata, Holothuroidea, *Paelopatides* sp.	1		1	1
Ophiuroid (white)	Echinodermata, Ophiuroidea sp1	2		2	2
Ophiuroid (red)	Echinodermata Ophiuroidea sp2	1		1	1
Crustacean	Arthropoda, Decapoda, *Probeebei mirabilis*	3		3	3
Salp (dead)	?	5		5	5
Polychaete	?	1	3	4	10
Crinoid	Echinodermata, Crinoidea	1	1	2	4
Isopod	Arthropoda, Isopoda, Munnopsidae	1	1	2	4
Bony fish	Chordata, Actinopterygii, *Ipnops* sp.	1		1	1
**Other anomalies**					
White exposed sediment		2	6	8	58
Green spot			9	9	56
White spot			20	20	131
**Identified/reference spectra**		**30**	**48**	**78**	**328**

Training spectra for the SVM classification were obtained from OOI identified based on video data and samples, and from additional spectral anomalies in the UHI images. SVM – Support Vector Machine.

Using the average spectra from the 78 identified OOIs as training spectra, the SVM method revealed a total of 328 OOIs across the 11 transects (Table 1). In general, all of the known OOIs were detected and classified correctly. In some cases, the marginal pixels of organisms were misclassified as other fauna categories with similar spectra, e.g. sponge instead of white coral, but the bulk part of the organism surface was classified correctly. This allowed each OOI to be clearly assigned to one of the previously defined categories.

**Figure 6:**
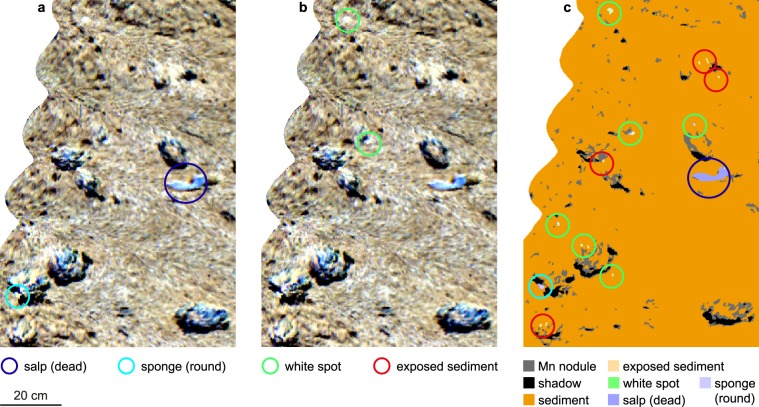
Illustration of the classification approach for a subsection of the UHI image in Fig. 5. (**a**) UHI image (in pseudo-RGB with R: 645 nm, G: 571 nm, B: 473 nm) showing a dead salp and a sponge that were identified from the video data. The sponge is the same that is shown in Fig. 1c. (**b**) Two further anomalies detected in the UHI image were assigned to the category “white spots” based on the spectral contrast angle, and potentially indicate the presence of chlorophyll *a*. (**c**) SVM classification image based on the training data in (**a**,**b**). The two megafauna were classified correctly, and in addition, other OOIs (white spots and sediment exposed from beneath the surface layer) were detected that had not been apparent from the video data.

In addition to the 78 known OOIs, 250 new OOIs were detected and classified by the SVM method (Table 1). On all transects, the number of OOIs revealed by the SVM results (6–71 OOIs) exceeded that of the OOIs identified in the video data (0–6 OOIs), showing that more anomalies were detected using the spectral classification approach than from analysis of the visual image data. This distribution is illustrated in Fig. 6 for a section of the track shown in Fig. 5. In this section, a dead salp and a sponge (Fig. 1c) identified from the video data were well apparent in the UHI image (Fig. 6a). Additional OOIs observed in the UHI image were identified as white spots based on the spectral contrast angle of the associated spectra (Fig. 6b). By including the average spectra from the identified OOIs in the training data, the SVM method correctly classified the training OOIs and revealed additional OOIs belonging to the white spot and exposed sediment categories (Fig. 6c).

Besides increasing the number of detected OOIs, the SVM approach was also able to detect OOIs with sizes on a sub-cm scale (down to 0.8 cm) and generally showed an increased detection rate for small (<2 cm) organisms such as small corals and polychaetes compared to the video-based OOI detection (Table 1). For larger megafauna, such as crustaceans, salps, and holothurians (7–15 cm in length), the SVM method did not reveal any additional individuals besides those already identified from the video data.

In total, 328 OOIs, i.e., spectral anomalies that differed from the background sediment and manganese nodules, were identified across the 72.3 m^2^ covered by the 11 survey tracks. Megafauna constituted 83 of these OOIs, the majority being white corals (39 individuals, or 0.54 m^−2^), polychaetes (10 individuals, or 0.14 m^−2^), and sponges (8 individuals, or 0.11 m^−2^). Of the 245 sediment anomalies, 187 were green or white spots, corresponding to 2.59 m^−2^.

## Discussion

Our results show that more megafauna individuals were detected and identified from the SVM classification results than from visual identifications in the video data. For example, only three white corals were observed by human observers in the video data, while 39 individuals were revealed by the spectral classification results. Similarly, only one polychaete was identified in the video data alone, whereas spectral classification revealed a total of 10 polychaetes across three different transects.

We note that it was not possible to verify the spectra-based identifications in all cases, due to the small sizes of most of the animals (<2 cm) and the associated difficulty in clearly resolving them in the video data. The OOI identifications based on the spectral contrast angle method are nevertheless assumed to be accurate, as the smallest spectral contrast angle of each OOI was close to 0° (1.75° on average) and therefore indicated a good match to one of the 30 reference spectra from the previously identified OOIs. For the SVM method, however, misclassifications cannot be excluded. The overall classification accuracy of the SVM results for this dataset was estimated to >90%^26^, suggesting that most OOIs, but not all, may have been classified correctly. This is a greater accuracy than is commonly produced from visual identification alone, given the inter-observer bias commonly reported from multi-observer studies^7^.

Despite this uncertainty associated with the SVM classification results, our results demonstrate that if a set of reference spectra from known megafauna exists, further individuals of the same fauna type can be identified *in situ* based on their spectral characteristics, or individual optical fingerprints. Although previous studies linked organism identifications based on spectral reflectance signatures to the pigment compositions of the organisms^16,18,24^, we show that identifications based on spectral characteristics are possible even if the pigment compositions are unknown. Consequently, additional sampling of marine organisms – a challenging procedure in the deep sea – and subsequent extraction of pigment information is not required. Our study thus extends the use of hyperspectral imaging as a taxonomic tool for marine organisms from *in vivo* applications^16,21^ to *in situ* surveys.

The high spatial resolution of the UHI image data (1 mm pixel size), combined with the spectral classification approach, is especially valuable for the detection of smaller fauna individuals (<2 cm in size). Examples include the tubed polychaetes, some of the sponges and less developed coral colonies in the current study, which were often difficult to detect or resolve in the lower-resolution video data (smallest resolvable size ca. 2 cm). By analyzing their spectral responses and applying spectral classification, the detection and identification rate for these organisms was increased considerably.

In contrast to smaller fauna, larger megafauna measuring about 4–15 cm in length, such as the adult crustaceans, ophiuroids, dead salps, and holothurians in the analysed dataset, were generally easy to detect both in the video data and in the UHI images, with the spectral approach not revealing any additional individuals to those already logged from the visual data by human observers. However, the UHI assessment, based on optical fingerprints rather than physical characteristics of the individual fauna, may well make the data more suitable for automated approaches, given the difficulty for semi-automated systems to be trained to recognize fauna of highly variable aspect or appearance^7,28^.

Even for the larger fauna, knowing the spectral responses of these may in some cases be used to indicate whether individuals belong to the same species. For example, in the case of the two holothurians observed, one was identified as belonging to the family Synallactidae based on the video data (Fig. 1f), while identification of the other holothurian was more difficult. This second individual (Fig. 1e) was interpreted as belonging to either Synallactidae or *Paelopatides* sp. Given that the spectral responses of these individuals differed markedly, especially in the red portion of the electromagnetic spectrum (Fig. 2c), they likely belonged to different species, and the second holothurian was therefore identified as *Paelopatides* sp.

Our results thus show that underwater hyperspectral imaging can be used as a non-invasive, *in situ* taxonomic tool for benthic megafauna. Even without knowledge of pigment composition and associated spectral absorption signatures, it is possible to differentiate marine organisms based on their unique spectral reflectance signatures in UHI images. Once a set of reference spectra based on identified organisms is established, spectral comparisons and application of supervised classification methods allow identification of benthic megafauna semi-autonomously without requiring additional sampling of animals. Especially in the deep sea, where sampling is difficult and time-consuming due to the high water depths, this approach is of great potential. For example, the assessment of the abundances of both small and larger megafauna may well be useful in gauging community recovery following a disturbance event in the deep sea. Aside from manganese nodule fields, deep-sea massive sulfide deposits and manganese crusts are economically attractive sources of deep-sea minerals^32–35^. Removal of these resources will wholly remove the colonized seafloor in these regions^11,36^, exposing new rock surfaces to the ocean. Monitoring the rate of post-exploitation colonization of these new surfaces by fauna will be an important component of management plans. The facility of underwater hyperspectral imaging to identify the early stages of colonization (i.e., the recently settled small corals, sponges, crinoids, etc.) could greatly aid in understanding faunal succession and relative success of any mitigation measures put in place in such areas^36^.

In addition to megafauna, the UHI is also able to detect and characterize seafloor anomalies with distinct spectral signatures. The distinct reflectance intensity minimum around 668–680 nm observed for the green and white spots suggests a possible relationship between these sediment anomalies and chlorophyll *a* concentration, which has a characteristic *in vivo* absorption maximum of around 675 nm^18,19,37,38^, or between 660–665 nm in *in vitro* studies^39^. Given the water depth of nearly 4200 m, the presence of chlorophyll *a* and associated biomass at the seafloor may seem unlikely. However, concentrations of chlorophyll *a* and its degradation products (phaeophytin/phaeophorbide *a*) have been observed at the abyssal seafloor in both the equatorial Pacific and the North Atlantic^40–43^. Chlorophyll *a* concentrations in these areas have been associated with phytodetritus deposited on the seafloor after seasonal phytoplankton blooms in the shallow water column^40–43^, with deposits accumulating either as continuous greenish layers or as cm-scale aggregates^42^.

Chlorophyll *a* concentrations have also been measured in seafloor sediments in the nearby DISCOL area, 700 m northwest of the study area (Matthias Haeckel, personal communication), although direct evidence for phytodetritus was not observed during the study period. However, at the time of the survey, a large fall of dead salp, fauna which bloom in upper waters in response to algal blooms, was ongoing^31^. Elevated chlorophyll *a* concentrations may possibly be related to this event. As the spectral characteristics of the green and white spots (Fig. 3) resemble those of degraded chlorophyll *a*, such as phaeophorbide *a* and/or phaeophytin *a*^44^, we interpret the green and white spots as indications for the presence of chlorophyll *a* or associated breakdown products, which may be related to the deposition of phytodetritus. However, physical samples from these anomalies would be required to confirm a relationship to chlorophyll *a* and its degradation products.

The difference between the green and the white spots is still unclear. One possibility is that the slightly different spectral responses reflect different chlorophyll *a* concentrations. Based on spectral reflectance studies in intertidal mud flats, Kromkamp *et al*.^38^ noted that an increase in biomass caused a decrease in reflectance, in particular around the chlorophyll *a* absorption maximum at 675 nm. As the green spots exhibit slightly lower spectral intensities than the white spots in this part of the electromagnetic spectrum (Fig. 3), the green spots may potentially be related to more biomass or higher chlorophyll *a* concentrations than the white spots. Chlorophyll *a* concentrations may be determined from reflectance spectra via a derived spectrometric index^19^, but this method could not be applied due to the availability of only pseudo-reflectance data, and hence chlorophyll *a* concentrations were not estimated.

Alternatively, the difference in reflectance intensity between the green and white spots could be due to a difference in decay stage of the phytodetritus. Reflectance of organic material may vary over time, as shown by Dierssen *et al*.^45^ in a study on floating wrack of the genus *Sargassum*. After an initial decrease of *Sargassum* reflectance over three days, reflectance increased again, with the effect of varying reflectance being most prominent around the chlorophyll *a* absorption maximum.

Most of the green and white spots with distinct spectral signatures in the UHI data are not apparent in the HD video data and would thus have been overlooked if only standard RGB data had been used for seafloor imaging. Moreover, the limited spectral information provided by the three RGB bands would not have allowed establishing a potential link to chlorophyll *a* based on the spectral responses. Although sampling is still required to confirm the presence of chlorophyll *a* or degradation products at the green and white spots, our results indicate that hyperspectral image data are well suited for the detection and imaging of chlorophyll *a* distribution on the seafloor, including in the deep sea. The facility of the approach to detect chlorophyll *a* at depth, remotely, and over extended areas is highly attractive, and may well support investigations into carbon transport pathways, particularly in remote regions with a high seasonality^46^. In addition to providing insight into the extent of seasonal phytodetritus deposition, mapping chlorophyll *a* distribution on the abyssal seafloor may also elucidate feeding habits of benthic megafauna, for which these surface deposits provide an additional food source^40–42^.

## Materials and Methods

### Data acquisition and processing

The current study used the UHI data collected and presented in Dumke *et al*.^26^ for location and identification of benthic megafauna and sediment anomalies. The data were acquired in a well characterized manganese nodule field in the Peru Basin (SW Pacific; 7°5′23″S, 88°26′46″W, 4195 m depth) during the RV SONNE cruise SO242/2, using a deep-sea UHI (UHI #4) by Ecotone AS (Trondheim, Norway) mounted on the KIEL6000 ROV (GEOMAR)^26,31^.

The UHI is a push-broom scanner with beamwidths of 60° (transverse) and 0.4° (longitudinal). It was mounted on the ROV’s outstretched manipulator arm and looked vertically downwards to record lines of 1600 pixels perpendicular to the track direction^26,31^. Seafloor illumination was provided by ten ROV light sources, which included an LED, halogen lamps (five Deep Multi-SeaLite lamps, two Sea Arc 5000 lamps), and two HMI lamps (SeaArc2). For each image pixel, the UHI recorded the intensities of the reflected light for 112 spectral bands between 378 nm and 805 nm with a spectral resolution of 4 nm.

UHI data were recorded throughout 11 ROV tracks across the seafloor, passing over various benthic fauna and manganese nodules (ROV dive SO242/2_191–1^31^). The tracks were run at a constant speed (0.05 m s^−1^) and heading, with track lengths ranging between 1.7 m and 20 m. The relatively constant altitude of 1–1.2 m resulted in a track width (field of view) of 1–1.2 m. In addition to the UHI data, HD and SD video data were simultaneously collected by the ROV cameras during each survey track.

As the spectral bands <400 nm and >710 nm were rather noisy, spectral subsetting was applied to the UHI data to reduce the data to the 83 bands between 400 nm and 710 nm. The raw UHI data contained several spectral components associated not only with the OOIs, but also with illumination, the inherent optical properties of the water column (scattering and absorption), vehicle motion, and imager properties^15^. These external influences had to be corrected for in order to obtain reflectance data in which the spectral response is specific to the OOIs.

For this initial deep-sea deployment of the system, it was not possible to completely correct for all external influences. The raw UHI data were first calibrated to radiance data by applying radiometric correction using the Hypermap software tool (Ecotone AS). To process the radiance data to true reflectance data, knowledge of the illumination influence (the combined spectral characteristics of the ROV lights for each image pixel location) and the inherent optical properties of the water column were required. As these characteristics could not be determined, they were approximated by a reference spectrum calculated from the UHI image data collected during each track. By diving each pixel spectrum by its reference spectrum, followed by smoothing of the spectra with a moving average filter for a window of 11 bands (44 nm), most external influences could be eliminated (for further information on data processing, see Dumke *et al*.^26^). However, as the corrected data still contained some residual influences, mainly from the illumination source and geometry, they represented “pseudo-reflectance” data rather than true reflectance data^26^.

The pseudo-reflectance data were of sufficient quality to allow differentiation between seafloor features and fauna based on the different optical fingerprints^26^, but they may not be fully comparable to true reflectance data. The corrected UHI data were georeferenced (for further details, see Dumke *et al*.^26^) and output with a pixel size of 1 mm. The UHI data used in this study are available from PANGAEA (https://doi.pangaea.de/10.1594/PANGAEA.874408).

### Data analysis

The analysis of the UHI data and the video data involved several steps. Firstly, the video data collected at the same time as the UHI data were analysed for the presence of OOIs, in this case megafauna individuals and any obvious sediment anomalies. These OOIs constituted the reference group. Animals were identified to the highest possible taxonomic level based on their appearance in the video data, and from previous physical sampling of megafauna in the area^30,31,47^. For each identified OOI, an average pixel spectrum was extracted from the UHI images. To account for any spectral variations or effects of bi-coloration across the OOI surface (such as overgrowth of portions of a surface by small encrusting sponges etc.), the investigation of which was outside the scope of this study, these pixel spectra were averaged over OOI surface areas of between 20 and 600 pixels, depending on the size of the OOI.

The UHI images were then analysed for additional OOIs that had not been identified from the video data and were associated with distinct spectral anomalies. Average pixel spectra from pixel areas of 20–200 pixels were extracted for these selected OOIs. To identify the OOIs, each OOI spectrum was compared against the 30 reference spectra from the OOIs identified from the video data by calculating the spectral contrast angle θ (Equation (1))^48^, which has to date primarily been applied in the comparison of mass spectra^48–51^ but is also applicable for determining similarities between any kind of spectra^48^.

1 $$cos\theta =\frac{{\sum }_{i}{a}_{i}{b}_{i}}{\sqrt{{\sum }_{i}{a}_{i}^{2}\,{\sum }_{i}{b}_{i}^{2}}}$$

where a_i_ and b_i_ correspond to the reflectance intensities of the two spectra a and b. The spectral contrast angle is a measure of the similarity of two spectra and ranges between 0° and 90°, where an angle of 0° means that the two spectra are identical in form^48^. For each unidentified OOI, θ was calculated for each reference spectrum and the OOI was subsequently assigned to the category associated with the most similar spectrum (smallest θ).

Following this step, spectral classification of the UHI images was performed using the ENVI software (v. 5.3; Exelis VIS). In spectral classification, each pixel spectrum is compared to training data, i.e., a set of known reference spectra from identified organisms or materials. The pixel spectra are then assigned to categories depending on which reference spectrum they match best^52,53^. Spectral classification was conducted using the Support Vector Machine (SVM) method, a standard classification technique often superior to other classification algorithms^54,55^ and known for its robustness with noisy and complex data^56^. The classification results were output as classification images showing the distribution of the different spectral categories.

This study used the same classification images presented in Dumke *et al*.^26^, but results and interpretations herein focus on benthic megafauna and sediment anomalies, features which were not discussed by Dumke *et al*.^26^. For spectral classification of the megafauna and sediment anomalies, the spectra of the previously identified OOIs were used as training data. The SVM classification results were then analysed to determine (i) if the training OOIs had been classified correctly, and (2) if any additional OOIs had been detected by the SVM method.
